# Retrospective analysis of the clinical efficacy of topical hyaluronic acid in promoting skin recovery and reducing inflammatory reactions after fractional laser treatment

**DOI:** 10.3389/fmed.2026.1778486

**Published:** 2026-05-05

**Authors:** Chungwen Yen, Tsean Yen

**Affiliations:** 1 Xiamen JiGuang Aesthetic Clinic, Xiamen, Fujian, China; 2 Department of Electrical Engineering, National Tsing Hua University, Hsinchu, Taiwan

**Keywords:** fractional laser, hyaluronic acid, inflammatory reactions, promoting skin recovery, topical hyaluronic acid

## Abstract

**Background:**

Fractional laser treatment is widely used to improve skin problems such as scars and photoaging; however, it is prone to causing adverse reactions such as erythema and edema after the procedure. Topical hyaluronic acid can provide moisturizing, reparative, anti-inflammatory, and healing effects, but related research remains limited.

**Objective:**

This study aimed to explore the effectiveness and safety of topical hyaluronic acid dressings in promoting skin recovery and reducing inflammatory reactions after fractional laser surgery, thereby providing a reference for clinical postoperative care.

**Method:**

This study is a retrospective comparative cohort study, with data collection and analysis conducted by researchers who did not participate in patient treatment. A total of 113 patients who underwent fractional laser treatment in our hospital from January 2021 to January 2025 were included. Patients were categorized into two groups based on their postoperative care regimen documented in medical records: the Routine Care (RC) group (*n* = 55) and the routine care + hyaluronic acid (HA) group (*n* = 58). The main observation indicators included patient’s skin recovery-related indicators (time to erythema disappearance and time to scab shedding) and levels of inflammatory factors [serum interleukin-6 (IL-6) and tumor necrosis factor alpha (TNF-*α*)]. The secondary observation indicators included skin barrier function indicators [transepidermal water loss (TEWL) and stratum corneum water content], melanin index (MI), erythema index (EI), and pain visual analog scale (VAS) scores, as well as monitoring of blood routine, liver and kidney function, and local adverse reactions. A multivariate regression analysis was used to explore independent factors affecting postoperative skin recovery, and the efficacy and safety of the comprehensive evaluation plan were evaluated.

**Results:**

The HA group had significantly shorter times to erythema disappearance and scab shedding than the RC group (*p* < 0.05). At postoperative days 7 and 14, the HA group exhibited lower serum IL-6 and TNF-*α* levels (*p* < 0.05). On day 14, it also had lower TEWL and higher skin moisture content (*p* < 0.05). Meanwhile, MI, EI, and VAS scores were also notably lower (p < 0.05). A multivariate regression analysis confirmed the use of topical hyaluronic acid as an independent favorable factor for shortening erythema regression time [odds ratio (OR) = 7.394, 95% confidence interval (CI): 2.353–23.240, *p* < 0.001] and reducing day-14 IL-6 levels (OR = 8.109, 95% CI: 3.327–19.768, *p* < 0.001). No between-group differences were found in blood routine, liver/kidney function, or adverse reaction incidence (*χ*^2^ = 0.004, *p* = 0.952).

**Conclusion:**

The combination of hyaluronic acid dressings with routine nursing care was associated with improved skin recovery and reduced inflammatory reactions after fractional laser surgery in this observational comparative cohort, suggesting potential clinical value for postoperative management.

## 1 Introduction

In the era of fast-evolving modern skin aesthetic medicine, laser treatment technology has been widely used in scar repair, photoaging improvement, and the treatment of pigmentary disorders, bringing significant cosmetic effects and improved quality of life to patients. Among them, fractional laser, as an innovative treatment modality, generates small thermal injury columns arranged in an array, which stimulate the self-repair mechanism of the skin while preserving the surrounding normal skin tissue, thereby promoting collagen remodeling and epidermal regeneration, and has become an important modality in skin rejuvenation treatment (1). Fractional laser technology includes two categories: non-ablative and ablative fractional laser modalities. The former stimulates collagen remodeling in the dermis through selective photothermal action, while the latter achieves more significant therapeutic effects by vaporizing the epidermis and some dermal tissues (2). However, despite the advantages of precise efficacy and a relatively short recovery time associated with fractional laser therapy, postoperative adverse reactions such as erythema, edema, pain, scab formation, and pigmentation commonly occur. These reactions not only affect the patient’s treatment experience but may also affect the final cosmetic outcome (3). Postoperative erythema is one of the most common early reactions to fractional laser therapy, typically ranging from several days to weeks. Its severity is closely related to laser energy density, treatment density, and individual skin sensitivity (4). In addition, laser thermal injury can activate the inflammatory cascade in the skin, leading to the release of various pro-inflammatory molecules and further exacerbating tissue edema and erythema (5).

Endogenous hyaluronic acid (HA) is a naturally occurring glycosaminoglycan within skin tissue, with strong moisturizing properties that promote wound healing and regulate inflammatory response with biological effects (6). As an ideal wound-healing agent, HA can promote skin repair through mechanisms such as regulating fibroblast proliferation, promoting collagen synthesis, and accelerating epithelial regeneration (7). At the same time, HA also has anti-inflammatory properties, exerting inhibitory effects on nuclear factor kappa B (NF-κB) pathway activation, thereby attenuating the synthesis of pro-inflammatory factors such as tumor necrosis factor alpha (TNF-*α*) and interleukin-6 (IL-6), ultimately alleviating the inflammatory response (8). However, the biological effects of HA, including its anti-inflammatory effects, are highly dependent on its molecular weight. While high-molecular-weight HA is typically anti-inflammatory and pro-homeostatic, low-molecular-weight fragments can be pro-inflammatory (9). Therefore, selecting the appropriate HA formulation is critical for therapeutic application. In terms of skin barrier repair, HA can increase stratum corneum hydration levels, reduce transepidermal water loss (TEWL), and improve skin barrier function (10). Over the past few years, the application of HA in aesthetic dermatology and wound repair has become increasingly widespread, and various HA dressings have been developed for postoperative care (11).

Despite the theoretical benefits, high-quality clinical evidence supporting the use of topical HA specifically after fractional laser surgery remains limited (12). Existing studies are often small-scale, lack objective assessments of inflammatory biomarkers and skin barrier function, or focus primarily on subjective patient feedback (12). Therefore, well-designed clinical trials are needed to objectively evaluate the efficacy and safety of HA in this specific postoperative context. This retrospective study was designed to systematically evaluate the efficacy of topical HA dressings in promoting skin recovery and reducing inflammation after fractional laser surgery. In alignment with HA’s established biological functions, the primary endpoints focused on clinical measures of tissue repair (time to erythema resolution and time to scab shedding) and inflammatory modulation (serum IL-6 and TNF-*α* levels). Secondary endpoints were selected to assess HA’s proposed effects on restoring skin barrier function (TEWL and stratum corneum hydration levels) and alleviating postoperative pain (VAS scores). By comprehensively evaluating these objective indicators, this study aims to provide a scientific basis for postoperative nursing strategies and to further elucidate the clinical value of HA in this specific context.

## 2 Methods

### 2.1 Study subjects

**Figure 1:**
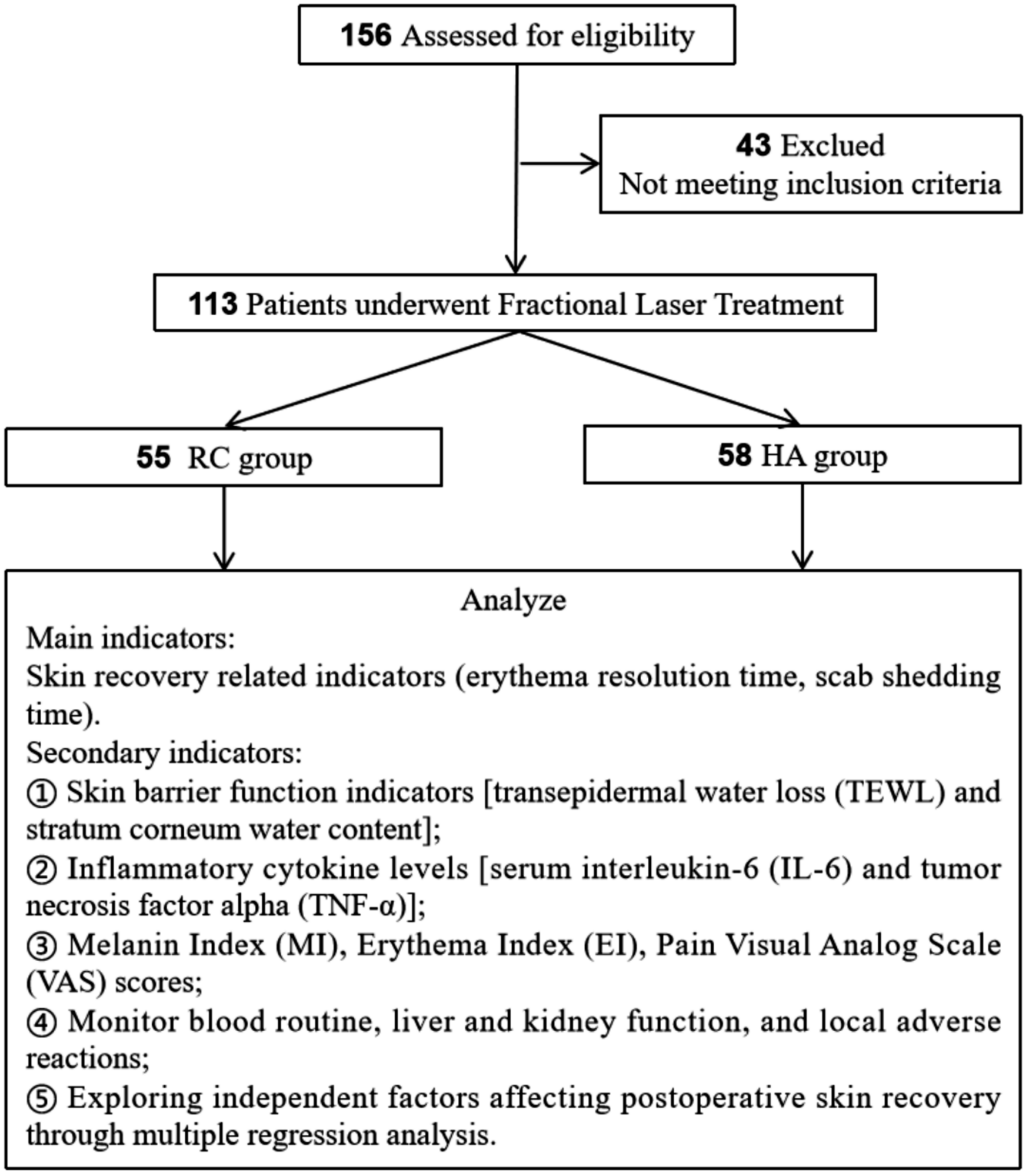
Research flowchart.

This was a retrospective comparative cohort study conducted at Xiamen JiGuang Aesthetic Clinic. Data were extracted from the medical records of patients who underwent fractional laser treatment between January 2021 and January 2025. The exposure (topical HA use) was determined by the attending physician’s clinical judgment and standard practice rather than randomization. The study followed the Strengthening the Reporting of Observational Studies in Epidemiology (STROBE) guidelines for reporting observational research. Ultimately, 113 patients were included in the statistical analysis. Patients who received routine postoperative cold compress repair care are divided into the RC group, while patients who received HA-enhanced cold compress in addition to routine care are divided into the HA group. Clinical indicators such as skin recovery, skin barrier function, and levels of inflammatory factors were compared between the two groups. The research flowchart is shown in Figure 1.

### 2.2 Inclusion criteria

The inclusion criteria were as follows: (1) age range: 18–65 years old; (2) treatment with ablative fractional laser (Fraxel CO₂ laser) for acne scars, photoaging, or pigmentation; (3) the treatment area was the face; (4) Fitzpatrick skin classification II–IV (13); (5) availability of complete clinical data before and after treatment; (6) follow-up time after surgery of at least 2 weeks.

### 2.3 Exclusion criteria

The exclusion criteria were as follows: (1) patients with active infections or skin diseases in the treatment area (13); (2) those with a history of keloid formation or poor wound healing; (3) pregnant or lactating women; (4) those who received other facial laser treatments or chemical peels within the past 3 months prior to treatment; (5) presence of autoimmune diseases or those undergoing immunosuppressive therapy; (6) those with severe heart, liver, and kidney dysfunction; and (7) those unable to cooperate in completing follow-up evaluation.

### 2.4 Ethical statement

This study strictly adheres to the principles of the Declaration of Helsinki (14), and all patient data were anonymized to ensure privacy protection. The research protocol was reviewed and approved by the Ethics Committee of Xiamen JiGuang Aesthetic Clinic (Approval No.: 2025-02-03; date of approval: 21 February 2025, Scope: Retrospective analysis of de-identified medical records from patients who underwent fractional laser treatment). The committee granted a waiver of written informed consent for this retrospective study. During the research process, patient privacy is protected, and all data were only used for scientific research purposes to ensure data security.

### 2.5 Sample size estimation

This study is a retrospective comparative cohort study. The study used an independent samples *t*-test for data analysis and G-power software for sample size calculation (15). According to previous studies (13), the selected effect size is 0.7, the significance level (*α*) is 0.05 (bilateral), and the statistical power (1-*β*) is 0.95. These parameters indicate that each group requires 45 participants, for a total of 90 participants. In the end, a total of 113 participants were included in this study, exceeding the target sample size and meeting the statistical requirements of the study.

### 2.6 Treatment measures

#### 2.6.1 Fractional laser treatment plan

All patients were treated with the same fractional laser device (Fraxel CO₂ Laser, Solta Medical, United States), with therapeutic parameters adjusted based on the patient’s lesion subtype, skin type, and treatment site: energy density of 10–15 mJ/cm ^2^, spot size of 100–300 μm, density of 100–200 dots/cm^2^, treatment frequency of one session, and treatment time of 15–30 min. Before treatment, the skin was routinely cleansed, and a compound lidocaine cream (5%) was applied for topical anesthesia. After 40 min, the cream was removed, the skin was disinfected, and the laser treatment was initiated.

#### 2.6.2 Postoperative nursing care plan

The postoperative care regimen (routine care alone or routine care with topical HA) was identified from medical records based on the attending physician’s clinical judgment and standard practice at the time of treatment. To assess potential selection bias, baseline demographic and clinical characteristics were compared between the two groups.

RC group (routine nursing group): Immediately after surgery, medical cold compress patches were applied for 30 min to relieve redness, edema, and pain. Within 1 week after surgery, patients were advised to avoid getting the wound wet and keep the skin clean and dry. Broad-spectrum sunscreen (SPF ≥ 50, PA+++) was applied topically twice daily with strict sun protection; and a basic moisturizer (containing ingredients such as ceramide and squalane) was used twice daily.

HA group (HA combined with routine nursing group): The routine nursing measures are the same as those of the RC group. On this basis, HA dressing (specification: each tablet contains 15 mg of HA, molecular weight: >1,000 kDa (high molecular weight); formulated in a sterile aqueous gel vehicle; national drug approval number H20140113, Shandong Bosilen Furuida Pharmaceutical Co., Ltd.) is applied topically 24 h after surgery. The method involved cleaning the skin and evenly applying the dressing to the treatment area for 30 min each time, twice daily. All patients received the same HA product. Given the non-randomized assignment based on clinical judgment, between-group comparisons were adjusted for potential confounders, as described in the statistical analysis section. Additionally, the HA group received structured application time (30 min, twice daily) beyond routine care, which may introduce co-intervention bias.

### 2.7 Observation indicators

All data were retrospectively extracted from existing medical records generated during routine clinical care between January 2021 and January 2025. Postoperative follow-up followed the clinic’s standard protocol: Patients were scheduled for routine visits at days 3, 7, and 14 (designated T1, T2, and T3 for analysis purposes), during which clinicians documented clinical outcomes as part of standard care. To ensure objectivity and minimize bias, all data collection and scoring were performed by two trained research assistants who were independent of the clinical treatment team and blinded to the patients’ group status (RC or HA). Clinical scores requiring subjective judgment (e.g., erythema resolution and scab shedding) were assessed independently by both assistants, and the average of their scores was used for analysis. Objective measurements (e.g., TEWL, MI, and EI) were obtained using standardized devices operated by a single trained technician. All measurements in this study were taken three times and averaged.

#### 2.7.1 Main observation indicators

(1) *Time to redness disappearance*: It is defined as the number of days required from the end of treatment to the complete disappearance of visible erythema. It was observed and recorded by the same dermatologist under natural light, the a* value in the International Commission on Illumination (CIE) Lab color system was used for quantitative evaluation. When the a* value returned to the preoperative baseline level, it was determined that the erythema had subsided (16).
(2) *Time to scab shedding*: It is defined as the number of days required from the end of treatment to complete scab shedding in all minor treatment areas. Two independent evaluators (blinded to group assignment) reviewed these existing clinical photographs to assess scab shedding. Standardized digital photographs were taken at each follow-up visit for routine medical record documentation and for tracking healing progress. These photographs are part of the patient’s clinical record, not taken solely for research purposes. Blinded assessment was performed by two independent trained nurses as part of our clinic’s internal quality assurance and complication monitoring system. These assessors are not involved in the patients’ direct care and are blinded to the postoperative care regimen as a standard practice to ensure objective documentation. Their assessments are recorded in the medical records for routine clinical use.
(3) *Inflammatory marker data*: Serum IL-6 and TNF-*α* levels are routinely measured at postoperative days 7 and 14 as part of our clinic’s inflammatory response monitoring protocol to detect potential excessive inflammation or infection early (measured by ELISA using commercially available kits, R&D Systems, United States). This is a standard practice for all patients receiving ablative fractional laser treatment. The assay was performed strictly according to the manufacturer’s instructions (17).

#### 2.7.2 Secondary observation indicators

(1) *Skin barrier function*: During routine postoperative evaluations, TEWL and stratum corneum hydration were measured using standard clinical devices (Tewameter® TM 300 and Corneometer® CM 825, Courage+Khazaka, Germany) as part of the standard clinical assessment protocol at this clinic (18). The measurement environment was stable thermo-humidity conditions (22 ± 2 °C, 50 ± 5% humidity), and patients rested quietly for 30 min before measurement.
(2) *Melanin and erythema indices*: Melanin and erythema indices were measured using a skin pigment analyzer (Mexameter® MX 18, Courage+Khazaka, Germany) on the day before surgery and 14 days after surgery (17).
(3) *Pain assessment*: Pain assessment was based on retrospectively collected visual analog scale (VAS) scores at 3 and 7 days postoperatively.
(4) *Adverse reactions*: Adverse reactions were defined as the retrospective collection of local adverse reactions within 30 days after surgery, as well as changes in blood routine and liver and kidney function before and after treatment.

All measurements were performed by a single trained technician following the device manufacturer’s guidelines. The calibration of the Tewameter®, Corneometer®, and Mexameter® was performed daily using standard reference materials before each measurement session. All readings were taken from three predefined anatomical sites on the treatment area (forehead, bilateral cheeks) and averaged to ensure reproducibility. The measurement environment was maintained under stable thermo-humidity conditions (22 ± 2 °C, 50 ± 5% relative humidity), and patients rested for 30 min before assessment.

### 2.8 Statistical analysis

To account for the non-randomized design, a multivariate regression analysis was performed, adjusting for potential confounders, including demographics, baseline clinical characteristics, and laser parameters. SPSS 25.0 was used for data analysis. Normally distributed quantitative data were expressed as mean ± SD, with inter-group comparisons performed using independent *t*-test and intra-group comparisons performed over time using repeated measures ANOVA. Non-normally distributed data were shown as M (Q1, Q3) and compared via the Mann–Whitney U test. The count data were presented as *n* (%) and were analyzed using the chi-square test. A *p*-value of <0.05 indicated statistical significance. Given the retrospective and exploratory nature of this study, no adjustment for multiple comparisons was applied across multiple endpoints and time points; therefore, the findings should be interpreted as hypothesis-generating.

## 3 Results

### 3.1 Comparison of baseline characteristics

Table 1 shows the characteristics of patients in the two groups undergoing fractional laser surgery in terms of sex, age, BMI, smoking status, alcohol consumption, skin type, preoperative TEWL, and indications. The results revealed no significant inter-group differences in baseline characteristics (*p* > 0.05), demonstrating satisfactory comparability prior to treatment.

**Table 1:** Baseline characteristics [mean ± SD, *n* (%)].

Variables	RC group (*n* = 55)	HA group (*n* = 58)	*χ^2^*/*t*	95% CI	*p*
Sex					
Male	23 (41.8)	22 (37.9)	0.178	-	0.673
Female	32 (58.2)	36 (62.1)			
Age (years)	32.4 ± 8.6	31.8 ± 9.1	0.361	−2.714, 3.924	0.719
BMI (kg/m^2^)	22.8 ± 2.1	22.6 ± 2.3	0.491	−0.605, 1.003	0.624
Smoking status	17 (30.9)	15 (27.3)	0.354	-	0.552
Alcohol consumption	13 (23.6)	13 (22.4)	0.024	-	0.877
Skin type (Fitzpatrick)					
II	12 (21.8)	15 (25.9)	0.306	-	0.858
III	28 (50.9)	29 (50.0)			
IV	15 (27.3)	14 (24.1)			
TEWL (preoperative)	17.6 ± 3.0	17.4 ± 2.6	0.372	−0.863, 1.262	0.711
Indication					
Acne scar	24 (43.6)	27 (46.6)	0.362	-	0.834
Photoaging	19 (34.5)	17 (29.3)			
Pigmentation	12 (21.8)	14 (24.1)			

BMI, body mass index; the same below.

### 3.2 Skin recovery status

Skin recovery status of the two groups is illustrated in Table 2, including the time to erythema disappearance and scab shedding. The values in the HA group were significantly shorter than those in the RC group (*p* < 0.05), suggesting an association between topical HA use and accelerated skin recovery in this cohort.

**Table 2:** Patient’s skin recovery status (mean ± SD).

Variables	RC group (*n* = 55)	HA group (*n* = 58)	95% CI	*t*	*p*
Time to redness resolution (days)	6.9 ± 1.5	5.3 ± 1.3	1.015, 2.028	5.951	<0.001
Time to scab shedding (days)	7.8 ± 1.7	6.5 ± 1.4	0.655, 1.730	4.395	<0.001

### 3.3 Comparison of skin barrier function

Table 3 and Table 4 shows the comparison of TEWL and skin moisture content between the two groups of patients. On the day before surgery, no notable inter-group difference was detected (*p* = 0.711). On the 7th and 14th postoperative days, the TEWL in the HA group was significantly lower than that in the RC group (*p* < 0.001), and skin moisture content levels in the HA group were significantly higher than those in the RC group (p < 0.001). Repeated-measures analysis showed time, inter–group, and interaction effects (all *p* < 0.05, *F* values listed) for TEWL and cutaneous moisture content. Intra-group comparisons vs. baseline (T0) were significant (p < 0.05), confirming superior skin barrier function in the HA group.

**Table 3:** Comparison of TEWL (mean ± SD, g/h/m^2^).

Time point	RC group (*n* = 55)	HA group (*n* = 58)	95% CI	*t*	*p*
T0	17.6 ± 3.0	17.4 ± 2.6	−0.863, 1.262	0.372	0.711
T2	29.9 ± 3.5	26.4 ± 3.3	2.334, 4.768	5.474	<0.001
T3	25.6 ± 3.4	22.4 ± 3.7	1.878, 4.521	4.797	<0.001
F	F-Time = 871.206	F-Group = 29.541		F-Time*Group = 23.982	
*P*	<0.001	<0.001		0.001	

**Table 4:** Comparison of skin moisture content (mean ± SD, AU).

Time point	RC group (*n* = 55)	HA group (*n* = 58)	95% CI	*t*	*p*
T0	57.1 ± 8.8	56.8 ± 9.3	−3.176, 3.581	0.119	0.906
T2	37.8 ± 6.7	45.5 ± 5.9	−10.049, −5.349	6.492	<0.001
T3	44.3 ± 5.4	52.7 ± 4.8	−10.364, −6.522	8.710	<0.001
F	F-Time = 103.104	F-Group = 54.554		F-Time*Group = 9.768	
*P*	<0.001	<0.001		0.001	

### 3.4 Comparison of inflammatory factor levels

Table 5 compares the levels of inflammatory factors in two groups of patients. There was no significant difference in baseline cytokine levels between the two groups of patients (*p* > 0.05). On postoperative days 7 and 14, the levels of IL-6 and TNF-*α* in the HA group were significantly lower than those in the RC group (*p* < 0.05), indicating that HA cold compress application after fractional laser surgery can significantly alleviate inflammatory reactions.

**Table 5:** Comparison of inflammatory factor levels (mean ± SD, pg./mL).

Variables	Time point	RC group (*n* = 55)	HA group (*n* = 58)	95% CI	*t*	*p*
IL-6	T0	5.8 ± 0.8	5.8 ± 0.7	−0.219, 0.317	0.361	0.719
	T2	33.5 ± 2.8*	31.6 ± 3.0*	0.8413, 2.801	3.683	<0.001
	T3	24.3 ± 3.5*	20.4 ± 3.1*	2.767, 5.228	6.438	<0.001
TNF-α	T0	6.1 ± 1.0	6.0 ± 1.2	−0.443, 0.376	0.163	0.871
	T2	49.2 ± 5.9*	42.3 ± 5.6*	4.757, 9.044	6.380	<0.001
	T3	38.4 ± 5.8*	31.1 ± 5.9*	5.118, 9.484	6.626	<0.001

* indicates that there is a significant difference in cytokine levels between T2 and T0, as well as between T3 and T0, and T3 and T2 (*p* < 0.05).

### 3.5 Comparison of MI and EI

Table 6 compares the melanin and erythema indices of two groups of patients before and 14 days after surgery. It was found that no notable inter-group difference was observed on the first day before operation (*p* = 0.277, *p* = 0.937). On the 14th day after surgery, the melanin and erythema indices of the HA group exhibited statistically lower measurements than the RC group (*p* < 0.001), implying that HA cold compress application after fractional laser surgery can significantly reduce the occurrence of melanin and erythema.

**Table 6:** Comparison of MI and EI (mean ± SD, AU).

Variables	Time point	RC group (*n* = 55)	HA group (*n* = 58)	95% CI	*t*	*p*
MI	T0	130.7 ± 12.9	133.5 ± 14.1	−7.847, 2.269	1.093	0.277
	T3	153.6 ± 13.6*	140.4 ± 13.4*	8.155, 18.221	5.192	<0.001
EI	T0	245.5 ± 19.9	245.3 ± 18.3	−6.838, 7.412	0.080	0.937
	T3	317.3 ± 15.2*	286.5 ± 15.4*	25.076, 36.502	10.679	<0.001

* indicates there is a significant difference in the comparison within the group compared to the level of T0 (*p* < 0.05).

### 3.6 Pain assessment

Table 7 compares VAS scores of two groups of patients at 3 and 7 days after surgery. VAS scores for the HA group were significantly lower than those of the RC group (*p* < 0.001), implying that HA cold compress application after fractional laser surgery can significantly reduce pain.

**Table 7:** Comparison of VAS score (mean ± SD).

Time point	RC group (*n* = 55)	HA group (*n* = 58)	*Z*	*p*
T1	4 (3, 4.5)	2 (2, 3)	4.932	<0.001
T2	2 (1, 2)*	1 (1, 1)*	4.102	<0.001

* indicates there is a significant difference in the comparison within the group compared to the level of T1 (*p* < 0.05).

### 3.7 Comparison of postoperative adverse reactions

Both groups of patients showed no significant abnormalities in blood routine, liver and kidney function tests, and pre- and post-treatment vital sign data. As illustrated in Table 8, three adverse reactions occurred in the RC group throughout treatment, including two cases of contact dermatitis (3.6%) and one case of pigmentation (1.8%), with an adverse reaction rate of 5.5%. There were two adverse reactions in the HA group, including one case of mild itching (1.7%) and one temporary exacerbation of erythema (1.7%), with an adverse reaction rate of 3.4%. No notable statistical discrepancy in adverse reaction rates was detected between the two groups (*χ*^2^ = 0.004, *p* = 0.952), with complete resolution of all events within 7 days of targeted care.

**Table 8:** Comparison of postoperative adverse reactions [*n* (%)].

Variables	RC group (*n* = 55)	HA group (*n* = 58)	*χ* ^2^	*p*
Contact dermatitis	2 (3.6)	0 (0)		
Pigmentation	1 (1.8)	0 (0)		
Mild itching	0 (0)	1 (1.7)		
Temporary exacerbation of erythema	0 (0)	1 (1.7)		
Overall	3 (5.5)	2 (3.4)	0.004	0.952

### 3.8 Multivariate logistic regression analysis

In the multivariate logistic regression analysis, the outcome of “quick recovery” was defined as erythema resolution time ≤ the median of the overall cohort (6 days), and “low inflammatory response” was defined as day-14 IL-6 ≤ the median of the overall cohort (22 pg./mL), based on the distribution of the data to facilitate clinical interpretability. Table 9 shows that sex, age, BMI, smoking status, alcohol consumption, skin type, preoperative TEWL, indications, and whether HA was used after surgery were included in the multivariate logistic regression model. The results showed that the use of HA (OR = 7.394, *p* < 0.001) was an independent favorable factor in shortening the time to erythema regression (model *χ*^2^ = 4.050, *p* = 0.853; Nagelkerke *R*^2^ = 0.242). Table 10 shows that when low IL-6 levels (≤22 pg./mL) 14 days after surgery were used as the dependent variable and the above indicators were included in a multivariate logistic regression model, the results showed that the use of HA (OR = 8.109, *p* < 0.001) is also an independent favorable factor in reducing IL-6 levels 14 days after surgery (model *χ*^2^ = 19.620, *p* = 0.012; Nagelkerke *R*^2^ = 0.301).

**Table 9:** Multivariate logistic regression analysis of quick recovery.

						95% CI for OR	
Variables	B	SE	Wald	*p*	OR	Lower limits	Upper limits
Hyaluronic acid used	2.001	0.584	11.724	0.001	7.394	2.353	23.24
Female	−0.33	0.527	0.392	0.531	0.719	0.256	2.02
Age ≤ 32 years	0.234	0.519	0.204	0.651	1.264	0.457	3.494
BMI ≤ 22	0.334	0.525	0.404	0.525	1.397	0.499	3.91
Smoking status	0.664	0.586	1.284	0.257	1.943	0.616	6.133
Alcohol consumption	0.803	0.586	1.88	0.170	2.232	0.708	7.033
Skin type (Fitzpatrick) III	0.539	0.674	0.639	0.424	1.715	0.457	6.427
Skin type (Fitzpatrick) IV	0.37	0.757	0.238	0.625	1.447	0.328	6.387
Preoperative TEWL ≤ 17	−0.072	0.526	0.019	0.891	0.931	0.332	2.61
Photoaging	−0.531	0.598	0.789	0.374	0.588	0.182	1.898
Pigmentation	0.284	0.609	0.217	0.641	1.328	0.403	4.38
Constant	−3.203	1.081	8.781	0.003	0.041	-	-

*χ*^2^ = 4.050; *p* = 0.853; Nagelkerke *R*^2^ = 0.242.

**Table 10:** Multivariate logistic regression analysis of low inflammatory response.

						95% CI for OR	
Variables	B	SE	Wald	*P*	OR	Lower limits	Upper limits
Hyaluronic acid used	2.093	0.455	21.196	<0.001	8.109	3.327	19.768
Female	−0.599	0.473	1.600	0.206	0.549	0.217	1.390
Age ≤ 32 years	0.136	0.464	0.086	0.769	1.146	0.462	2.845
BMI ≤ 22	−0.080	0.462	0.030	0.862	0.923	0.374	2.281
Smoking status	0.250	0.532	0.220	0.639	1.284	0.453	3.639
Alcohol consumption	0.255	0.534	0.228	0.633	1.291	0.453	3.678
Skin type (Fitzpatrick) III	−0.576	0.569	1.022	0.312	0.562	0.184	1.717
Skin type (Fitzpatrick) IV	0.243	0.680	0.127	0.721	1.275	0.336	4.830
Preoperative TEWL ≤ 17	−0.115	0.490	0.055	0.815	0.892	0.341	2.330
Photoaging	0.133	0.515	0.067	0.796	1.142	0.416	3.133
Pigmentation	−0.061	0.567	0.012	0.914	0.940	0.310	2.857
Constant	−0.514	0.770	0.446	0.504	0.598	-	-

*χ*^2^ = 19.620, *p* = 0.012; Nagelkerke *R*^2^ = 0.301.

## 4 Discussion

Fractional laser induces controllable microthermal damage, stimulates the skin’s wound-healing response, promotes collagen remodeling and epidermal regeneration, and has become an effective means of treating skin problems such as acne scars and photoaging (19). Numerous clinical studies have corroborated that both 10,600 nm-wavelength carbon dioxide (CO_2_) lattice laser and Er:YAG laser with a 2,940 nm emission wavelength can effectively improve moderate-to-severe atrophic acne scars, with an overall efficacy of over 70% and good safety (20). However, this invasive treatment inevitably damages the structure and triggers a brief but significant inflammatory response, manifested as acute phase symptoms such as postoperative erythema, edema, pain, scabbing, and increased TEWL (21). These adverse reactions not only affect patients’ treatment tolerance and quality of life but may also prolong the recovery period and increase the risk of post-inflammatory hyperpigmentation (PIH), particularly in populations with darker skin tones (22). Therefore, optimizing postoperative nursing strategies to accelerate recovery and reduce adverse reactions is a key focus of clinical attention. As this was a retrospective cohort study with non-randomized group assignment, the findings should be interpreted as associations rather than causal effects.

This study found the HA group had significantly shorter time to erythema resolution and scab shedding than the RC group (*p* < 0.05), linked to HA’s wound-healing role. HA is not only a key component of the extracellular matrix but also a dynamic signaling molecule that regulates various cellular behaviors by interacting with its main receptors, CD44, RHAMM, etc. Based on previous *in vitro* studies, exogenous HA, particularly medium-molecular-weight fragments, can promote cell migration and proliferation by activating the PI3K/Akt/mTOR signaling pathway in epidermal keratinocytes, which is crucial for rapid re-epithelialization of small wounds after laser surgery (23). Consistent with this finding, we observed faster re-epithelialization in the HA group, suggesting that similar mechanisms may have contributed to the accelerated recovery in our patients. In addition, HA may elevate the expression levels of tight junction proteins such as occludin and claudin-1 in keratinocytes, which helps to quickly rebuild the integrity of the epidermal barrier in the early stage of re-epithelialization (24), reduce TEWL, and block external stimuli. This may partially explain the faster erythema disappearance observed in the HA group in this study. In addition, HA’s excellent moisturizing ability can create an ideal “moist healing” environment for wounds. This environment has been proven to reduce the formation of hard crusts in necrotic tissue, maintain cellular activity, and serve as a reservoir for various growth factors, thereby accelerating the healing process (25). Therefore, the faster shedding of scabs observed in this study is likely the consequence of a synergistic action of HA through the aforementioned multiple mechanisms.

Laser thermal injury activates keratinocytes, fibroblasts, and immune cells, leading to the substantial release of key pro-inflammatory mediators (TNF-*α*, IL-1β, and IL-6), mediating early erythema, edema, and pain (24). Pro-inflammatory factors such as IL-17A (26) can also mediate inflammatory responses in autoimmune diseases. In this study, we observed a sustained decrease in inflammatory cytokine levels in the HA group, which may reflect the effective regulation of this local and systemic inflammatory microenvironment by topical HA, laying a molecular foundation for reducing postoperative redness and swelling and accelerating repair. High-molecular-weight HA is typically viewed as a steady-state signaling molecule, while low-molecular-weight fragments (produced by inflammation or injury) have pro-inflammatory potential (27). This provides a key molecular mechanism explanation for the observed decrease in serum inflammatory cytokine levels in this study. However, exogenous supplementation of specific molecular weight HA can intervene and reshape this pathological process. Although our clinical data do not directly elucidate the underlying molecular mechanisms, we hypothesize that topically applied HA may modulate the post-laser inflammatory microenvironment through several pathways supported by previous research. For instance, research has shown that locally applied HA can inhibit the interaction between endogenous pro-inflammatory LMW-HA fragments and cell surface receptors, such as TLR4 and CD44, through competitive binding mechanisms (28). This competitive inhibition can effectively block the excessive activation of the NF-κB signaling pathway, ultimately leading to a significant reduction in the synthesis and secretion of pro-inflammatory cytokines. NF-κB acts as a critical signaling pathway in the inflammatory phase of wound healing, whose core role is to activate B cells and induce the expression of pro-inflammatory cytokines such as TNF-*α*, IL-1, IL-6, and IL-8 (29). In traumatic environments, HA can facilitate the conversion of macrophages from pro-inflammatory M1 type to anti-inflammatory reparative M2 type (30). M2 macrophages release anti-inflammatory factors such as IL-10 and transforming growth factor-*β*, promoting fibroblast activation and matrix remodeling (31, 32). This study observed a sustained decrease in inflammatory cytokine levels in the HA group, which may reflect the effective regulation of this local and systemic inflammatory microenvironment, laying a molecular foundation for reducing postoperative redness and swelling and accelerating repair (33).

Skin barrier function essentially relies on the “brick wall structure” of the stratum corneum and its sebum membrane (34). Laser treatment can temporarily damage this structure, leading to a sharp increase in TEWL and lowered skin hydration content. At 14 days postoperatively, the HA group had significantly lower TEWL and higher skin moisture than the RC group, indicating HA effectively accelerates skin barrier recovery. The superior barrier recovery observed in the HA group is likely attributable to a combination of physical and biological effects. At the physical level, HA may produce a breathable hydrophilic coating on skin, directly reducing water evaporation and providing sustained hydration, which is particularly important in the early postoperative period (35). At the biological level, HA has a regulatory effect on the expression of barrier-related proteins (36). A 2023 study using reconstructed human epidermal models found that treatment with HA-containing formulations significantly increased the content of natural moisturizing factor (NMF), a degradation product of filaggrin, in the model epidermis (37). NMF is a key water-absorbing substance in the corneal layer, and its increased content directly enhances the skin’s water-holding capacity. In addition, as mentioned earlier, HA promotes the expression of tight junction proteins, which helps to strengthen the barrier function of the epidermal granular layer and spinous layer and reduce secondary inflammation caused by irritant penetration (38). Therefore, the rapid recovery of barrier function in patients with HA is a comprehensive manifestation of HA’s dual effects of external water locking and internal enhancement of the stratum corneum structure and function.

Postoperative pain and burning sensation are common subjective adverse reactions of the fractional laser. HA group patients exhibited markedly lower VAS scores than their RC group counterparts at 3 and 7 days after surgery. In this study, the peak period of acute inflammation and pain was observed at 3 days postoperatively (T1), during which the release of inflammatory factors and neural stimulation were most significant, with a focus on immediate relief of discomfort. This node only has clear inter-group differences in VAS scores, which precisely confirms that HA rapidly reduces peak pain through physical soothing and early anti-inflammatory measures; the remaining indicators have not yet formed significant differences due to the acute impairment of the skin barrier and the lack of apparent repair effects. In addition to its anti-inflammatory effect, which indirectly reduces the stimulation of nerve endings by inflammatory mediators such as prostaglandins and bradykinin, HA may also alleviate pain through more direct mechanisms (39). The excellent moisturizing and cooling effects of HA, especially when used in the form of cold compress patches, can provide immediate physical relief. A humid environment can reduce the sensitivity of nerve endings, while low temperature itself has the effect of constricting blood vessels and slowing down nerve conduction velocity, effectively alleviating postoperative burning and stabbing sensations. The improvement of subjective comfort is of great significance for enhancing patients’ overall satisfaction and compliance with treatment.

Post-inflammatory pigmentation is a complication that needs to be monitored after fractional laser surgery, particularly in Fitzpatrick III-IV skin, and its phenomenon is closely linked to the degree of inflammatory reaction and the activity of melanocytes (36). MI and EI were significantly lower in the HA group than in the RC group at 14 days post-surgery. This discovery has significant clinical implications. First, as mentioned earlier, HA effectively inhibits inflammatory factors such as IL-6 and TNF-*α*, thereby reducing the stimulation of melanocytes from the source. IL-6 and other factors can activate microphthalmia transcription factor (MITF), upregulate tyrosinase expression, and promote melanin synthesis. Second, emerging research suggests that HA itself may have a direct regulatory effect on the melanin production pathway (40). In addition, by accelerating barrier repair and reducing erythema, HA shortens the time when the skin is in a fragile and inflammatory state, indirectly reducing the risk of pigmentation induced by environmental factors such as ultraviolet radiation. Therefore, the lower MI and EI in the HA group in this study are a positive result of the combined effects of HA anti-inflammatory action, its possible direct inhibition of melanin synthesis, and its role in promoting overall repair.

## 5 Study limitations

This study is a retrospective comparative cohort study, and although data collection was conducted by researchers who did not participate in the treatment, there are still certain limitations. First, the effectiveness under different settings (e.g., regions and equipment parameters) needs to be validated. Second, although the sample size meets statistical requirements, further expanding the sample size may improve research effectiveness. Third, the molecular weight of HA has not been stratified, and there may be differences in the biological activity of HA with different molecular weights. Fourth, the follow-up period is only 2 weeks, lacking long-term efficacy and safety data, making it impossible to assess the effect of HA on long-term pigmentation and scar improvement. Additionally, the non-randomized design introduces potential selection bias, and the additional structured application time in the HA group may have contributed to a time-and-attention bias that cannot be fully separated from the treatment effect. These limitations should be considered when interpreting the results. Subsequent studies are recommended to conduct multicenter, large-scale, prospective randomized controlled trials to optimize the study design. Further exploration can be conducted on the differences in therapeutic effects of HA with varying molecular weights and concentrations, as well as the specific molecular mechanisms underlying its effects. Meanwhile, the follow-up period should be extended to 3–6 months to evaluate long-term effectiveness and safety. In addition, imaging techniques such as dermoscopy and confocal microscopy can be used to dynamically observe the histological effects of HA on dermal collagen remodeling and epidermal repair.

## 6 Conclusion

In summary, the combination of HA dressing and routine care after fractional laser surgery can significantly promote skin recovery, reduce inflammatory reactions, improve skin barrier function, reduce pigmentation and erythema, alleviate postoperative pain, and demonstrate a favorable safety profile. In this observational comparative cohort, the use of HA dressing alongside routine care was associated with improved postoperative outcomes. These findings support further investigation in prospective randomized controlled trials to establish causality and to isolate the specific effects of HA from those of co-interventions.

## Data Availability

The original contributions presented in the study are included in the article, further inquiries can be directed to the corresponding author.
